# Individual Genomic Loci, Transcript Levels, and Serum Profiles of Immune and Antioxidant Markers Associated with Bacteria-Induced Abortion in Sheep (*Ovis aries*)

**DOI:** 10.3390/vetsci12080719

**Published:** 2025-07-31

**Authors:** Attia Eissa, Ahmed A. Elsayed, Amin Tahoun, Adel M. El-Kattan, Islam M. Wassif, Amani A. Hafez, Ragab Mohamed, Ahmed I. Ateya, Hanan M. Alharbi, Khairiah M. Alwutayd, Aiman A. Ammari, Manal A. Babaker, Mansour A. Alghamdi, Torsten Bohn, Ammar AL-Farga, Hossam M. Aljawdah

**Affiliations:** 1 Department of Animal Medicine (Internal Medicine), Faculty of Veterinary Medicine, Arish University, Arish 45511, Egypt; attia.ahmed@vet.aru.edu.eg; 2 Department of Animal Health and Poultry, Animal and Poultry Production Division, Desert Research Center (DRC), Cairo 11753, Egypt; sahara.seha@drc.gov.eg (A.M.E.-K.); islam.wassif@drc.gov.eg (I.M.W.); amani.hafez@drc.gov.eg (A.A.H.); 3 Department of Clinical Veterinary Medical Sciences, Jordan University of Science and Technology, Irbid 302022110, Jordan; amtahoun@just.edu.jo; 4 Department of Animal Medicine, Faculty of Veterinary Medicine, Kafrelshkh University, Kafrelsheikh 33516, Egypt; 5 Department of Theriogenology, Faculty of Veterinary Medicine, Aswan University, Aswan 81528, Egypt; drragab@vet.aswu.edu.eg; 6 Department of Development of Animal Wealth, Faculty of Veterinary Medicine, Mansoura University, Mansoura 35516, Egypt; 7 Department of Biology, College of Science, Princess Nourah bint Abdulrahman University, P.O. Box 84428, Riyadh 11671, Saudi Arabia; hmalharbi@pnu.edu.sa (H.M.A.); kmalwateed@pnu.edu.sa (K.M.A.); 8 Zoology Department, College of Science, King Saud University, P.O. Box 2455, Riyadh 11451, Saudi Arabia; aammari@ksu.edu.sa; 9 Department of Chemistry, Faculty of science, Majmaah University, Al Majmaah 11952, Saudi Arabia; m.babaker@mu.edu.sa; 10 Department of Anatomy, College of Medicine, King Khalid University, Abha 62529, Saudi Arabia; m.alghamdi@kku.edu.sa; 11 Genomics and Personalized Medicine Unit, The Center for Medical and Health Research, King Khalid University, Abha 62529, Saudi Arabia; 12 Nutrition and Health Research Group, Population Health Department, Luxembourg Institute of Health, 1A-B, Rue Thomas Edison, L-1445 Strassen, Luxembourg; 13 Department of Biological Sciences, College of Science, University of Jeddah, Jeddah 21589, Saudi Arabia; amalfarga@uj.edu.sa; 14 Department of Zoology, College of Science, King Saud University, P.O. Box 2455, Riyadh 11451, Saudi Arabia; haljawdah@ksu.edu.sa

**Keywords:** antioxidants, immunity, single-nucleotide polymorphisms, gene expression, abortion and sheep

## Abstract

Abortion is a major cause of production losses in sheep. This study aimed to investigate the genetic susceptibility and serum profile of immune and antioxidant markers associated with bacterial abortion in Aboudeleik ewes. A total of 80 animals were included: 43 healthy controls and 37 aborted ewes. Clinical samples (blood, placenta, vaginal swabs, and aborted fetuses) were subjected to bacteriological culture, PCR, gene expression analysis using qRT-PCR, SNP detection via DNA sequencing, and serum biochemical profiling. The predominant bacteria isolated from aborted fetuses included *Brucella melitensis*, *Salmonella* spp., and *Campylobacter* spp. Gene expression analysis revealed significant upregulation of immune-related genes (TLR4, IL-8, IL-17, NF-kB) and downregulation of antioxidant genes (SOD3, CAT, Nrf2) in aborted ewes. DNA sequencing identified 25 single-nucleotide polymorphisms (SNPs), including 10 non-synonymous SNPs associated with abortion susceptibility. Biochemical analyses confirmed increased levels of pro-inflammatory cytokines and oxidative stress markers and decreased levels of IL-10, antioxidant enzymes, progesterone, and IFN-τ in aborted ewes. These findings suggest that distinct genomic and immunological profiles are associated with abortion susceptibility and may serve as potential biomarkers for selective breeding and disease management in sheep.

## Simple Summary

This study investigated individual genomic loci, transcript levels, and serum profiles of immune and antioxidant markers associated with bacteria-induced abortion in Aboudeleik sheep. The bacteria isolated from abortion ewes and fetuses were *Brucella melitensis*, *Salmonella* sp., and *Campylobacter* sp. The study revealed distinct changes in immunological and antioxidant indicators in terms of nucleotide sequence and gene expression between healthy and aborted ewes. These verdicts provide insight into the pathogenesis of abortion and support future disease control and breeding strategies.

## 1. Introduction

Aboudeleik sheep are a prominent native breed in desert and semi-desert areas, known for their adaptability to harsh climates and their utility in both milk and meat production [1]. They typically exhibit a lean body structure with relatively long limbs and an average body weight of approximately 45 kg [1]. Common physical traits include elongated heads and necks, often featuring a skin fold (dewlap), besides a long tubular tail that extends beyond the hocks. The ears are characteristically small or absent and are oriented downward and backward. Their coat usually presents a mix of dark hues such as black or brown, blended with lighter cream tones [2].

Sheep and goats are essential to the daily profits of rural populations, particularly in low-income countries, where they serve as vital sources of income, nutrition, and social investment. Most of these animals, nearly 90%, are raised in remote areas with limited access to advanced farming technologies or veterinary support. As a result, reproductive challenges and decreased productivity in small ruminants are commonly attributed to disease exposure and poor management practices [3].

Pregnant ewes are particularly vulnerable to abortion, which results in large financial losses for small-scale livestock farmers. It is a factor that limits output, since it increases the number of infertile females kept in the flock for prolonged periods of time and reduces the possible number of replacement stocks for flock and milk production [3,4]. Sheep and goat abortions are frequently the result of multiple factors. Variation in management parameters such as health care, feeding and watering methods, and nutrition management for pregnant animals has an impact on the survival of the fetus. Additionally, seasonality, agroecological variables, and production systems all have a major impact on the prevalence of abortion [5]. Infectious and non-infectious reasons can be used to classify abortion causes in general [6,7]. Management, diet, genetics, environment, and even combinations of these are among the non-infectious reasons [8,9]. Bacteria like *Campylobacter* spp., *Brucella melitensis*, *Listeria* spp., *Salmonella* spp., *Coxiella burnetii*, and *Chlamydia abortus*; viruses like bluetongue virus, plague of small ruminants virus, and Border disease virus; and parasites like *Toxoplasma gondii* or *Neospora caninum* are the most commonly found infectious causes of abortion in sheep [10,11]. These infections are also zoonotic and thus can represent major infection concerns for farming populations [12,13]. It takes a lot of time and effort to identify the bacterial pathogens that cause abortions since it is difficult to isolate the organisms [14]. For quick diagnostic needs, molecular diagnostic methods like PCR are suggested as a good substitute tool [15].

Blood biochemical investigations offer a wealth of information regarding an animal’s nutritional status, overall health, and well-being; hence, they can be used to assess an animal’s general state of health [16,17]. Certain blood parameters can be observed to deviate from normal limits; this can aid in the differential diagnosis of disorders and provide information on the extent of infection and tissue damage [18].

Numerous physiological processes, including oocyte maturation, fertilization, embryo growth throughout pregnancy, appropriate parturition, and the onset of premature labor, are impacted by oxidative stress, according to [19]. Through enzymatic induction and activity of superoxide dismutase (SOD), glutathione peroxidase (GPx), and glutathione (GSH), as well as nonenzymatic free-radical protectors and scavengers, plasma-free radical trapping and antioxidant potential in a normal pregnancy can counteract oxidative stress [20].

The induction and control of the immunological inflammatory response are significantly influenced by cytokines. They serve as inflammatory mediators of pro- and anti-inflammatory actions as well as positive or negative cell growth factors [21]. Cytokines exert a great influence on the type, strength, and length of the immune response through regulation of cell growth and maturation, as well as their activation, proliferation, and differentiation [22,23]. Additionally, they play a significant role in controlling the local immune response. Moreover, they impact the systemic acute phase response by influencing the hypothalamus–hypophysis–adrenal gland axis and other elements of the neuro-endocrine system [24].

By increasing animal health, cutting-edge molecular genetic approaches may be used as supplements to help control disease [25]. Several genetic markers, predominantly single-nucleotide polymorphisms (SNPs), have been successfully linked to disease susceptibility/resistance in livestock [26,27,28]. This shows that there are variances between host genomes in the degree of susceptibility/resistance to the disease [29]. Reproductive losses due to abortion and perinatal mortality are major constraints on livestock productivity, particularly in small ruminant production systems. Among the various contributing factors, infectious diseases, including brucellosis, salmonellosis, campylobacteriosis, and listeriosis, are responsible for significant fetal losses, decreased fertility, and increased culling rates. These conditions not only impact the health and welfare of animals, but also result in substantial economic losses for farmers and breeders.

Reproductively, Aboudeleik sheep are considered seasonal breeders, with the most natural breeding occurring during the autumn and winter months due to the decreasing photo period. However, under improved or semi-intensive systems, partial seasonal breeding has been observed. Moreover, Aboudeleik ewes typically demonstrate a strong maternal instinct, characterized by attentive mothering, efficient nursing behavior, and high lamb survival rates under extensive grazing conditions [30].

Despite these favorable traits, reproductive efficiency in Aboudeleik sheep remains suboptimal, largely due to the infectious causes of abortion. While some studies have examined the prevalence of abortion-related pathogens in small ruminants, limited data exist on the molecular mechanisms and genetic susceptibility underlying abortion in this breed. In particular, the role of host immune and antioxidant gene expression, as well as relevant gene polymorphisms, remains poorly understood. This study was therefore designed to investigate the microbiological, biochemical, and genetic factors associated with abortion in Aboudeleik ewes. We aimed to identify the major bacterial pathogens involved, assess the expression of key immune and oxidative stress genes, and explore associated SNP polymorphisms and serum biomarkers. By integrating these data, the study seeks to advance our understanding of the multifactorial nature of ovine abortion and contributes to improved reproductive management and disease control strategies in this economically important breed.

## 2. Materials and Methods

### 2.1. Study Area

This study was conducted at the Ministry of Agriculture and Land Reclamation’s Shalateen Research Station, the Desert Research Center (DRC), in the Red Sea Governorate’s Ras Hadraba Valley and Halaib region (approximately 1400 km southeast of Cairo, Egypt). The region is situated at latitudes 21°59′795″ and 22°59′992″ and longitudes 36°52′676″ and 36°45′002″. The area is categorized as desert, with the Red Sea to the east and Sudan to the south as its borders. Average ambient air temperatures are 35 °C and 22 °C, respectively, and summer and winter relative humidity values are 37% and 43%, respectively. About 58.5 mm of precipitation falls on average each year, primarily in the form of sporadic showers in November and December. Water resources are scarce, and the only people who have access to them are the nomadic people and their animals through shallow wells and seawater desalination. As a result, there are no agricultural operations, and the farmers’ only source of income is from their animals (sheep, goats, and camels) grazing on rangelands [31]. *Panicum turgidum* is the predominant plant species throughout the region in both the dry and wet seasons, according to [32,33]. It also produces the most fresh and dry fodder throughout both seasons [34].

### 2.2. Animals and Study Design

All animal procedures, including clinical examinations and biological sampling from both aborted and control ewes, were conducted in accordance with national guidelines for the care and use of animals in research. The study protocol was reviewed and approved by the Institutional Animal Care and Use Committee (IACUC) of the Desert Research Center, Cairo, Egypt, under approval number DRC-IACUC-031-1-23. Informed consent was obtained from livestock owners prior to sample collection.

This study was conducted on 80 clinically examined Aboudeleik ewes, including 37 animals with a recent history of abortion and 43 healthy controls. All animals were reared under semi-extensive systems in Shalateen as a desert area of Egypt, with the Red Sea to the east and Sudan to the south as its borders. The animals were maintained under comparable nutritional and management conditions throughout the study. The ewes were aged between 2 and 5 years, with an average parity of 2.8 ± 0.5, meaning each ewe had previously given birth approximately two to four times. The healthy control group included animals with no history of reproductive disorders, while the abortion group consisted of ewes that had experienced one or more abortions, either in the current or previous breeding season. Aboudeleik sheep are known for moderate fertility and fecundity, with reported conception rates exceeding 85% and lambing rates of 1.2 to 1.5 lambs per ewe annually, often resulting in single or twin births. These reproductive indicators, however, can be negatively influenced by environmental stressors and infectious disease challenges such as those explored in this study. The field and laboratory phases of the research began in September 2022 and continued through May 2023, encompassing a complete breeding and lambing cycle and spanning a total of nine months. This duration allowed for continuous clinical monitoring, timely sampling post-abortion, and comparison with control animals at matched gestational stages. All clinical assessments, blood collections, and fecal, placental, or vaginal sampling were performed within 24 to 48 h following abortion events and at equivalent gestational periods in controls. The collected samples were then subjected to bacteriological, molecular, biochemical, and gene expression analyses, as described in the following sections.

This study adopted a field-based, case control design to investigate associations between abortion status and various molecular, genetic, and biochemical markers. The animals in both the control and abortion groups were of the same breed (Aboudeleik sheep) and selected from the same research facility, ensuring uniformity in housing, nutrition, environmental conditions, and veterinary management to minimize external variability. Inclusion criteria for the abortion group required a confirmed history of abortion within the current reproductive cycle. The control group included control ewes with normal reproductive history. All animals underwent thorough clinical examination, and samples were collected using standardized, sterile protocols to ensure consistency across the study. Though formal matching was not applied, the enrolled ewes were similar in age (3.5 ± 0.3 years) and body weight (48 ± 2.2 kg), and all had experienced at least one previous lambing without complications. These measures minimize confounding due to management and physiological status. However, unmeasured confounders such as parity variation or latent infections may still exist and are acknowledged as a study limitation.

The sample size (80 ewes: 43 healthy and 37 aborted) was based on availability and ethical constraints during the natural lambing season. Post hoc power analysis was conducted using GPower 3.1.9.7. Assuming a two-tailed *t*-test, α = 0.05, and an effect size of 0.8, this sample size provides > 85% power to detect significant differences in gene expression. For SNP frequency comparisons using chi-square tests, the power exceeded 80% for variants with moderate to large effect sizes and allele frequencies above 20%.

### 2.3. Blood Sampling and Measurements

Blood was drawn from each ewe via jugular venipuncture using sterile 10 mL vacutainer tubes. Sampling occurred once per animal, timed between 24 and 48 h after abortion in affected ewes, and at equivalent gestational periods for control animals to maintain physiological consistency and minimize potential confounding effects due to gestational timing on progesterone and other hormonal markers. To control for diurnal influences on biochemical and hormonal levels, all samples were collected early in the morning, from 7:00 to 9:00 AM, before feeding. This standardized timing helped reduce variability related to circadian fluctuations. Collected blood was split into two fractions: one left to clot at room temperature and then centrifuged at 3000× *g* rpm for 10 min to obtain serum for biochemical and hormonal assays; the other was placed into EDTA-containing tubes and frozen at −80 °C for later molecular and gene expression testing. The samples were either analyzed immediately or stored under appropriate conditions to preserve their integrity until batch processing.

### 2.4. Bacteriological Examination

A total of 111 samples were taken from 37 aborted ewes, consisting of 37 aborted fetuses, 37 vaginal swab samples, and 37 placental swabs. Strict aseptic procedures were used to collect the samples so that a bacteriological analysis could be performed. Swabs were taken from the liver, spleen, and lungs, in addition to various samples of the stomach and intestinal contents of fetuses that were aborted. The swabs were collected into separate sterilized containers. Using sterile cotton swabs, the placenta and vaginal discharge were removed from the abortion group in a sterile way. The samples were sent to the lab for a bacteriological examination right away.

The samples were immediately inoculated onto blood agar, S.S. agar, blood-free selective agar, and Albimi agar plates. The plates that were injected with Albimi agar were incubated at 37 °C in an aerobic environment in jars or an incubator that contained 5–10% CO_2_. The *Campylobacter* blood-free selective agar, which includes antibiotics, was placed in a plastic anaerobic container. After that, the jar was kept in microaerophilic settings at 37 °C for three to four days, with reduced concentrations of 5% O_2_, 10% CO_2_ (produced with a CO_2_ generating kit), and 85% N2. Using the techniques outlined by [35], the likely Brucella colonies were recognized based on their morphology, staining reactions, and biochemical features. The identification of *Campylobacter* colonies was carried out using the procedures provided by [36]. For the course of 24 to 48 h, the plates holding the preceding four media were kept in an incubator at 37 degrees Celsius. Then, colonies thought to be interesting were chosen and streaked over a nutrient agar slant. After that, the slant was incubated for 24 h at 37 degrees Celsius to produce a culture free of contamination. The Gram stain reactions, biochemical features, and morphology of the putative colonies were used to identify them using the techniques outlined by [37].

In instances where the primary culture exhibited multiple colonies, individual colonies were carefully chosen based on distinct morphological traits such as size, coloration, hemolytic activity, and colony margins. These selected colonies were then streaked separately onto nutrient agar plates to isolate pure cultures. Each isolate underwent independent identification through Gram staining, biochemical characterization, and PCR assays, as applicable. Mixing different colony types was avoided to maintain the accuracy of pathogen identification and to ensure the detection of possible co-infections.

All procedures involving *Brucella melitensis* cultures were carried out in a Biosafety Level 3 (BSL-3)-certified laboratory at the Veterinary Serum and Vaccine Research Institute, Abbasia, Cairo, Egypt. The work complied fully with applicable national and international biosafety guidelines. Staff members were trained in BSL-3 protocols, and strict containment, handling, and disposal measures were enforced to prevent laboratory-acquired infections and environmental contamination.

### 2.5. Bacterial PCR Detection

The QIAamp DNA Mini kit (Qiagen, Hilden, Germany, GmbH) was used to extract DNA from the samples, with certain changes made in accordance with the manufacturer’s instructions. In summary, 200 µL of the sample suspension was treated for 10 min at 56 OC with 10 µL of proteinase K and 200 µL of lysis buffer, and 200 µL of 100% ethanol was added to the lysate following incubation. After that, the sample was centrifuged and cleaned in accordance with the manufacturer’s instructions. An elution buffer containing 100 µL was used to elute the nucleic acid.

A 25 µL reaction comprising 12.5 µL of EmeraldAmp Max PCR Master Mix (Takara, Hyogo Prefecture, Japan), 1 µL of each primer at a concentration of 20 pmol, 4.5 µL of water, and 6 µL of DNA template was used to use the primers. A heat cycler called Appliedbiosystem 2720 was used to carry out the reaction. The oligonucleotide primers used were supplied by Metabion, planegg, Germany) and are listed in Table 1. The concentration and purity of the extracted DNA were measured using a NanoDrop™ 2000 spectrophotometer (Thermo Scientific, Waltham, MA, USA), assessing absorbance at 260 nm and 280 nm. DNA purity was evaluated by calculating the A260/A280 ratio, with acceptable values ranging between 1.8 and 2.0. Additionally, the integrity of the DNA was confirmed by electrophoresis on a 1.5% agarose gel stained with ethidium bromide and visualized under UV light.

The PCR products were separated by electrophoresis employing gradients of 5 V/cm on a 1.5% agarose gel (Applichem, Darmstadt, Germany, GmbH) in 1x TBE buffer at room temperature. Twenty microliters of the items were put into each gel slot for gel analysis. Gelpilot 100 bp plus ladder (Qiagen, Hilden, Germany, GmbH) and Generuler 100 bp ladder (Fermentas, Frankfurt, Germany) were used to determine the fragment sizes. A gel documentation system (Alpha Innotech, Biometra, Kasendorf) took pictures of the gel, and computer software was used to analyze the data.

For each PCR run, both positive and negative controls were incorporated. Positive controls included DNA from known reference clinical strains or vaccine strains carrying the target genes. A no-template control (water) was used to check for any contamination during the amplification process. To confirm successful DNA extraction and exclude the possibility of PCR inhibitors, selected negative clinical samples were supplemented with control DNA and subjected to repeat testing. The positive controls consistently generated the expected amplification products, thereby validating the negative results obtained from the test samples.

### 2.6. Total RNA Extraction, Reverse Transcription, and Quantitative Real Time PCR

The candidate immune and antioxidant genes analyzed in this study were selected based on their established roles in inflammation and oxidative stress, particularly in relation to bacterial infection and reproductive health. The immune-related genes (TLR4, IL-8, IL-17, NF-kB, CFH, TMED1, ICAM1, SMURF1, and CSIFR) were chosen for their involvement in pathogen recognition, cytokine signaling, and immune cell activation, while the antioxidant markers (SOD3, CAT, Nrf2, Keap1, PRDX2, and HMOX1) were included due to their relevance to redox balance and cellular defense during pregnancy. Selection was informed by previous studies on small ruminants and cattle [45,46,47], as well as preliminary expression profiling in a pilot cohort.

The Trizol (Toronto, ON, Canada) reagent was used to extract total RNA from sheep blood in accordance with the manufacturer’s instructions (RNeasy Mini Kit, Catalogue No. 74104, Waltham, MA, USA). Using a NanoDrop^®^ ND-1000 Spectrophotometer, the amount of isolated RNA was measured and certified. The manufacturer’s protocol (Thermo Fisher, Catalogue No. EP0441 Waltham, MA, USA) was followed in the synthesis of the cDNA for every sample. Quantitative real-time RT-PCR was used to evaluate the gene expression configuration for coding fragments of immune (TLR4, IL-8, IL-17, NF-kB, CFH, TMED1, ICAM1, SMURF1, and CSIFR) and antioxidant (SOD3, CAT, Nrf2, Keap1, PRDX2, and HMOX1) genes using SYBR Green PCR Master Mix (2x SensiFast^TM^ SYBR, Bioline, CAT No. Bio-98002, London, UK).

Using the Quantitect SYBR green PCR kit ((Toronto, ON, Canada; catalogue number 204141), real-time PCR was used to measure the relative amount of mRNA. As indicated in Table 2, primer sequences were created in accordance with the *Ovis aries* sequence published in PubMed.

For normalization, the housekeeping gene ß. actin was employed as a constitutive control. In all, 3 µL of total RNA, 4 µL of 5x Trans Amp buffer, 0.25 µL reverse transcriptase, 0.5 µL of each primer, 12.5 µL 2x Quantitect SYBR green PCR master mix, and 8.25 µL RNase-free water made up the 25 µL reaction mixture. Upon placing the final reaction mixture in a thermal cycler, the following protocol was executed: 30 min of reverse transcription at 50 °C, 10 min of primary denaturation at 94 °C, 40 cycles of 94 °C for 15 s, 1 min of annealing temperatures as indicated in Table 2, and 30 s of 72 °C. A melting curve analysis was carried out at the conclusion of the amplification stage to verify the PCR product’s specificity. Using the 2^−ΔΔCt^ technique, the relative expression of each gene in each sample was determined in relation to the *ß. actin* gene [48].

All primers used in qRT-PCR were validated for amplification efficiency and specificity. Standard curves were constructed using 10-fold serial dilutions of pooled cDNA, and efficiency values ranged from 90% to 105%, with R^2^ values ≥ 0.98. Melt curve analysis confirmed the specificity of amplification for each primer set, with single peaks and no primer dimers detected. Due to the involvement of only two groups (healthy and aborted ewes), the housekeeping gene *β-actin* was selected as the internal control. Expression stability was assessed using NormFinder software, which showed a stability value < 0.15 across healthy and aborted groups, supporting its use for normalization.

### 2.7. DNA Sequencing and Polymorphism Detection

Before DNA sequencing, primer dimers, nonspecific bands, and other impurities were removed. As described [49], purification of real-time PCR products with the expected size (target bands) was carried out using a PCR purification kit following the manufacturer’s procedures (Jena Bioscience # pp-201×s, Munich, Hamburg, Germany). Quantification of the PCR product was carried out using Nanodrop (Uv-Vis spectrophotometer Q5000, Waltham, MA, USA) in order to yield high products and to ensure enough concentrations and purity of the PCR products [50]. To detect SNPs in genes investigated in control and aborted ewes, PCR products with target bands were sent for DNA sequencing in forward and reverse directions using an ABI 3730XL DNA sequencer (Applied Biosystem, Waltham, MA USA), depending on the enzymatic chain terminator technique developed by [51].

Analysis of DNA sequencing data was carried out using chromas 1.45 and blast 2.0 software [52]. Differences were classified as single-nucleotide polymorphisms (SNPs) between the PCR products of the investigated genes and the reference sequences available in GenBank. Using the MEGA6 version 6.0, software tool, variations in the amino acid sequence of the genes under investigation between the enrolled ewes were carried out based on the data alignment of DNA sequencing [53].

### 2.8. Biochemical Analysis

To measure the serum concentrations of pro-inflammatory cytokines (IL-1α, IL-1β, IL-6, TNF-α), anti-inflammatory cytokines (IL-10), insulin-like growth factor binding proteins (IGFBP1), and IFNT using RayBiotech VR ELISA kits, Waltham, MA the following commercial kits were utilized in accordance with the usual protocols of the providers. Biodiagnostic, Cairo Egypt, CAT No. MD2529, CA252417, GSH2511, NO 2533, and GP 2524, respectively, measured malondialdehyde (MDA), catalase (CAT), reduced glutathione (GSH), nitric oxide (NO), and glutathione peroxidase (GPx). A commercial test kit from Oxford Biomedical Research, Waltham, MA, USA was used to measure progesterone (Ref: EA74). All ELISA measurements, including IL-1α, IFN-τ, and other serum markers, were performed in duplicate for each sample according to the manufacturer’s protocols. Assay performance was monitored using pooled internal control sera. The intra-assay and inter-assay coefficients of variation were ≤10% and ≤12%, respectively, in accordance with the manufacturer’s specifications and confirmed across runs.

### 2.9. Control Procedures and Materials Used

To ensure the validity and reproducibility of the results, appropriate control materials and procedures were employed throughout all stages of the study. In the bacteriological assays, standard reference strains of *Brucella melitensis*, *Salmonella* spp., and *Campylobacter* spp. (ATCC strains) were used as positive controls to verify the culture conditions and confirm biochemical test reactivity. Negative controls included sterile swabs and uninoculated media plates, which were incubated in parallel with test samples to monitor potential contamination. Each biochemical identification assay was validated using known positive and negative strains.

For molecular and gene expression analyses, multiple layers of controls were applied. All qPCR and RT-qPCR reactions included no template controls (NTCs) to detect reagent contamination and no reverse transcriptase (No-RT) controls to rule out genomic DNA contamination. *GAPDH* was used as a housekeeping gene for normalization of expression data across all samples. RNA integrity was confirmed prior to cDNA synthesis by measuring A260/A280 ratios using a spectrophotometer and visualizing intact ribosomal bands on agarose gel electrophoresis.

In the biochemical and hormonal assays, ELISA kits, Waltham, MA were used according to the manufacturers’ protocols, including the application of internal quality controls, standard curves, and blank wells. All serum samples were analyzed in duplicate to ensure the consistency of measured values. These measures collectively ensured the technical rigor, biosafety, and reproducibility of the data obtained in this study.

### 2.10. Statistical Analysis

An independent samples *t*-test (SPSS software program, version 20, Chicago, IL, USA) was used to determine the differences in the biochemical parameters and gene expression of tested enzymes among animals of different disease statuses (healthy and aborted groups). The values were expressed as mean ± SD. Differences in the frequencies of each gene SNP between the aborted and control groups were statistically evaluated using the chi-square test. To correct multiple testing across the 25 SNPs, the Benjamini–Hochberg false discovery rate (FDR) method was applied, and adjusted *p*-values were calculated using SPSS version 23. Statistical significance was set at FDR-adjusted *p* < 0.05. A linear discriminant analysis (LDA) was conducted to determine whether gene-level SNP averages could differentiate between aborted and healthy ewes. The 15 gene average scores served as predictor variables, and the health status (abortion vs. healthy) was the grouping variable. Statistical significance was set at *p* < 0.05.

## 3. Results

### 3.1. Clinical Findings

The control group during normal lambing and postpartum stages exhibited normal feed intake, stable body temperature, absence of uterine discharge, and normal under condition. In contrast, the abortion group presented with clinical signs, including fever, loss of appetite, exhaustion, straining, elevated tail carriage, reddish-brown vaginal discharge, protrusion of fetal membranes, mammary gland development, and the presence of fetus or fetal membranes in the pen.

### 3.2. Bacterial Prevalence and Identification

Table 3 summarizes the bacterial isolates recovered from aborted fetuses, vaginal swabs, and placentas of aborted ewes. Out of 111 samples per site, 26 (23.4%) were positive for bacterial isolation. The predominant bacteria isolated from aborted fetuses were *Brucella melitensis* (13.5%), *Salmonella* spp. (10.8%), and *Campylobacter* spp. (8.1%). From vaginal swabs, *B. melitensis* was isolated at 8%, while *Salmonella* spp. and *Campylobacter* spp. were each isolated at 2.7%. Placenta samples yielded *B. melitensis* (10.8%), *Salmonella* spp. (8.1%), and *Campylobacter* spp. (5.4%). No isolates of *Listeria monocytogenes*, *Coxiella burnetii*, or *Chlamydia psittaci* were recovered from any samples.

Table 4 presents the distribution of bacterial isolates within different fetal organs. All isolated bacteria (*B. melitensis*, *Salmonella* spp., and *Campylobacter* spp.) were detected at 100% prevalence in stomach contents. Isolation rates decreased in the liver and spleen, with incidence ranging from 66.6% to 80%, and were lowest in the lungs (33.3% to 60%).

**Figure 1:**
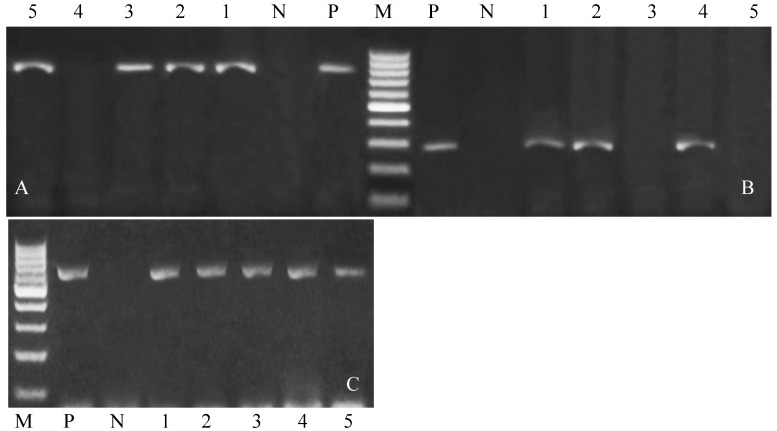
(**A**–**C**) Electrophoresis analysis of PCR product of amplified (**A**) *Brucella melitensis*, (**B**) *Salmonella* spp., and (**C**) *Campylobacter* spp. (**A**) Lanes 1, 2, 3, and 5 indicate a positive amplification of *Brucella melitensis* at the 839 bp, (**B**) Lanes 1, 2, and 4 indicate positive amplification of *Salmonella* at the 284 bp, and (**C**) all samples indicate positive amplification at 650 bp for *Campylobacter*.

**Figure 2:**
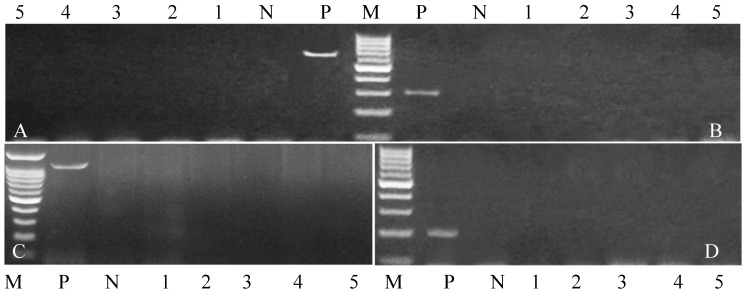
(**A**–**D**) Electrophoresis analysis of PCR product of amplified (**A**) *Coxiella burnetii*, (**B**) *Chlamydia psittaci*, (**C**) *Listeria monocytogens,* and (**D**) *Leptospira*. All sample negative for all bacteria at 687, 300, 1200 and 202 bp, respectively.

PCR analysis targeting five representative isolates (*Salmonella* spp., *Campylobacter* spp., *Listeria monocytogenes*, *Coxiella burnetii*, *Brucella melitensis*, and *Chlamydia psittaci*) confirmed the specificity of oligonucleotide primers by successful amplification of expected fragment sizes: 284 bp, 650 bp, 1200 bp, 687 bp, 839 bp, and 300 bp, respectively (Figure 1 and Figure 2) (original Figures S1 and S2). Notably, *Listeria monocytogenes*, *Coxiella burnetii*, and *Chlamydia psittaci* were negative in all tested samples.

### 3.3. Patterns for Transcript Levels of Immune and Antioxidant Indicators

**Figure 3:**
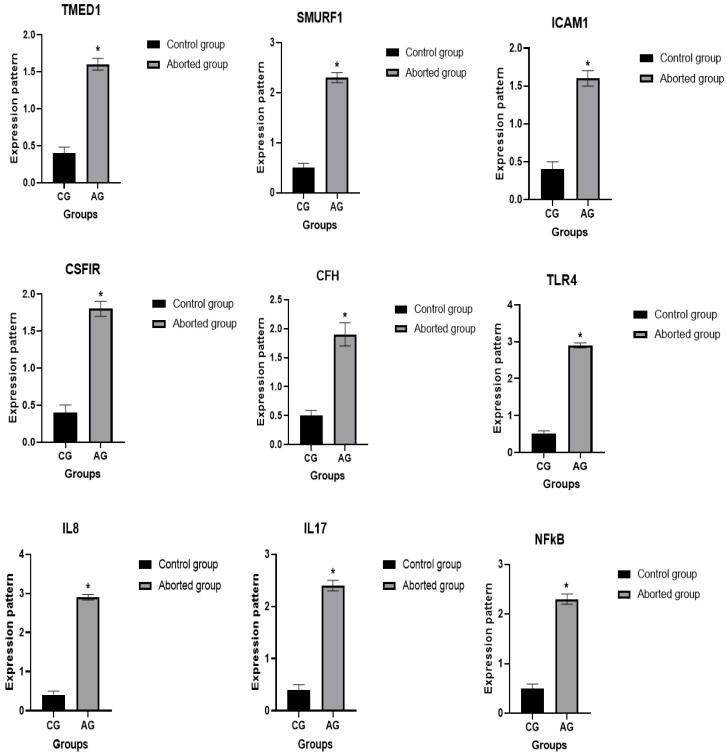
Comparative analysis of immune-related genes in normal and abortion-affected ewes. Values are mean ± SE. The symbol * denotes statistical significance (* *p* < 0.05).

**Figure 4:**
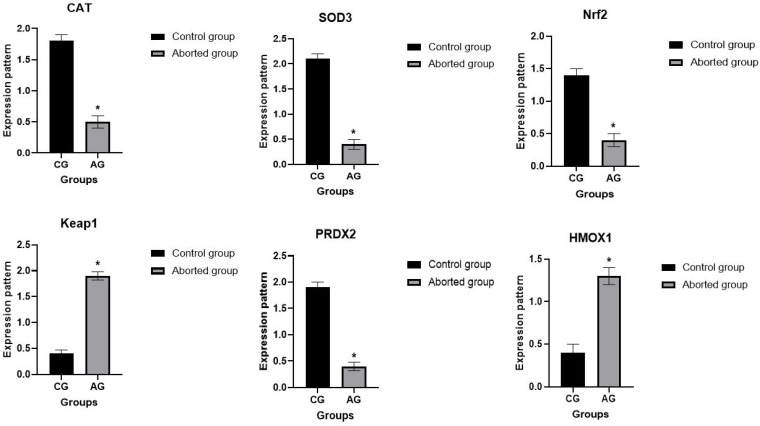
Comparative analysis of antioxidant gene expression in healthy and abortion-affected ewes. Values are mean ± SE. Asterisks (*) indicate statistically significant differences (* *p* < 0.05).

Figure 3 and Figure 4 illustrate the gene expression profiles of the selected immune and antioxidant markers. A significant difference was detected in the frequencies of all examined gene SNPs among the aborted and control groups (FDR-adjusted *p* < 0.05). The total chi-square value showed significant variation among the identified SNPs in all genes between the resistant and affected animals. The FDR correction confirmed the robustness of these associations, with all previously significant SNPs maintaining statistical significance after adjustment. Compared to healthy ewes, the abortion group showed significant upregulation of *TLR4* (*p* = 0.000), *IL-8* (*p* = 0.002), *IL-17* (*p* = 0.001), *NF-kB* (*p* = 0.001), *CFH* (*p* = 0.008), *TMED1* (*p* = 0.001), *ICAM1* (*p* = 0.002), *SMURF1* (*p* = 0.001), *CSF1R* (*p* = 0.001), *Keap1* (*p* = 0.006), and *HMOX1* (*p* = 0.007). Conversely, *SOD3* (*p* = 0.001), *CAT* (*p* = 0.001), *Nrf2* (*p* = 0.003), and *PRDX2* (*p* = 0.001) were significantly downregulated in aborted ewes. Among the aborted groups, *TLR4* exhibited the highest mRNA expression (2.99 ± 0.08), while *PRDX2* had the lowest (0.46 ± 0.09). In the healthy controls, *SOD3* showed the highest expression (2.14 ± 0.14), whereas *CSF1R* had the lowest (0.41 ± 0.11).

### 3.4. Genetic Polymorphisms of Immune and Antioxidant Genes

PCR amplification and sequencing of immune and antioxidant genes revealed multiple-nucleotide variations between aborted and healthy ewes. Fifteen synonymous and ten non-synonymous single-nucleotide polymorphisms (SNPs) were identified across the examined genes (Table 5 and Table 6). These SNPs are localized within exonic regions, resulting in coding sequence alterations in the abortion group relative to the controls. Statistical analysis showed significant differences in SNP frequencies between the groups (FDR-adjusted *p* < 0.05). Chi-square tests confirmed the distribution of SNPs differed significantly between aborted and healthy animals (*p* < 0.05). The FDR correction confirmed the robustness of these associations, with all previously significant SNPs maintaining statistical significance after adjustment.

Discriminant analysis assessing the correlation between health status and gene SNPs (Table 7) demonstrated 100% correct classification of animals, indicating the high discriminatory power of these genetic markers for predicting susceptibility to abortion. To evaluate the robustness of the discriminant model and assess potential overfitting, we applied Leave-One-Out Cross-Validation (LOOCV) to the dataset. The cross-validated classification accuracy was 92.5%, with 3 false positives and 3 false negatives, indicating that the SNP-based model maintained high predictive performance under rigorous validation. These findings support the discriminative potential of the selected SNPs while also acknowledging model limitations.

### 3.5. Biochemical Profile of Inflammatory and Antioxidant Markers

Serum levels of oxidative stress markers and cytokines in the control and abortion groups are summarized in Table 8 and Table 9. Compared to the controls, the abortion group showed significantly elevated levels of pro-inflammatory cytokines IL-1α, IL-1β, IL-6, TNF-α, malondialdehyde (MDA), nitric oxide (NO), and insulin-like growth factor binding protein 1 (IGFBP1) (*p* < 0.05). Conversely, anti-inflammatory cytokine IL-10, antioxidant enzymes catalase (CAT), glutathione peroxidase (GPx), glutathione reductase (GSH), progesterone, and interferon tau (IFN-τ) were significantly decreased (*p* < 0.05).

## 4. Discussion

This study explored the interplay between infectious agents, host genetic variation, and physiological responses in relation to abortion in Aboudeleik sheep. Our results showed clear associations between specific bacterial pathogens and significant changes in gene expression and serum biomarkers. These findings support the initial hypothesis that both immune-related genetic polymorphisms and oxidative stress responses play key roles in abortion susceptibility.

### 4.1. Clinical Discrimination Between Healthy and Aborted Sheep

There are many infectious and non-infectious causes of abortion in small ruminants. Veterinarians have long advised against this condition, yet it can still have serious financial repercussions due to lower meat and milk production, decreased milk yield, and decreased animal fertility [54,55]. A major zoonotic disease that has a major impact on public health and causes huge financial losses for small ruminant farms is brucellosis. *Brucella melitensis* is the primary cause of this illness in sheep and goats, and the primary sign of the ailment is abortion [56].

### 4.2. Bacterial Identification in Aborted Sheep

Regarding the bacteriological isolate, the most common microbe recovered from aborted sheep was *Brucella* spp., with an incidence rate of 10.8%. These findings were consistent with those of [57], whose authors found 10.4% of cases of Brucella in India, and [58], whose authors found 11.53% of cases in Iraq. These rates are notably lower than those found in other studies carried out in Iran (17.8%) [59] and in Konya Province, Central Anatolia [60], where Brucella was detected in aborted fetuses with an incidence of 31%; these studies were conducted in Egypt, with an incidence of 21.4% [61], 15.87% [62], 13.5% [47], and 18.9% [63], with the latter successfully isolating *Brucella melitensis* from aborted lambs.

According to [64], the rates using vaginal swabs in previous studies conducted in Iran (62.5%) were significantly greater than those reported by [65] in Algeria (5.32%). The number of pathogens, the disease stage, and the demanding qualities of the *Brucella species* all influence the likelihood of infection; harmful bacteria are expelled through body discharge [61]. Disease transmission is influenced by a number of variables, including geographic location, exposure level, reproductive health, technological developments in diagnosis, vaccination strategies, and the execution of national eradication programs [66].

The complex configuration of virulence factors can augment salmonella’s ability to cause systemic infection and, consequently, induce miscarriage in a variety of animal species. *Salmonella enterica* can get through the placental barrier and infect the developing fetus. Animals born prematurely or soon after birth may suffer from diseases, stillbirths, and abortions as a result [67]. *Salmonella* spp. have been known to induce abortion in cattle and small ruminants; this was reported by [68].

The incidence rate of isolated Salmonella spp. was in close agreement with those of [61,69], whose authors found 5.8% and 6.2% of *Salmonella* spp. in Egypt and the Mongolian National Statistical Office, respectively. The incidence rate, however, was lower by 8.89%, 11%, 12%, 15.87%, and 26.66% than those reported in Egypt by [62,70,71]. Furthermore, a prevalence rate of 34.3% was observed by [72] in Jordan, which was notably greater than the 3.2% reported by [73] in Ethiopia.

The frequency of *Campylobacter* spp. isolations was in close agreement with that of [69], whose authors identified *Campylobacter species* at a frequency of 4.8% in Egypt. These findings, however, surpass those of [61], which showed a prevalence of 7.1% in Egypt. The percentage of *C. fetus* isolated in Egypt was 1.4%; this is less than the percentages reported by [13] in Australia (32%) and [74] in Hungary (78.3% caused by *C. fetus* subsp. fetal and 21.7% caused by *C. fetus* subsp. *venerealis*). China too has a 70% isolation rate reported by [75].

The results of PCR, which was used to confirm the presence of *Brucella melitensis*, *Salmonella* sp., and *Campyl-obacter* sp. and detect *Listeria monocytogens*, *Coxiella burnetii*, and *Chlamydia psittaci,* were aligned with the information provided by [76]. In Lower Saxony, Germany, [77] found 3.7% and 3.3% of *Coxiella burnetii* and *Listeria monocytogenes*, respectively. In Turkey, 2.9% of people had both illnesses, according to [78].

Using PCR, [79] found that *C. abortus* was present in cattle fetuses that had aborted. The study found that the positive rate was 6.3%. By using PCR to examine the stomach contents, [80] found that 3% (2 out of 65) of aborted cattle fetuses in the Konya region of Turkey had *C. abortus*. The prevalence rates of *C. abortus* in Tunisia were found to be 6.6%, 1.9%, 3.2%, and 6.6 by [81,82,83,84], respectively.

### 4.3. Immune and Antioxidant Gene Polymorphism Linked with Abortion

Our results revealed expression and nucleotide sequence variants in the immune and antioxidant genes in healthy and aborted ewes. Few studies have examined the link between the candidate gene approach and abortion incidence in sheep. The polymorphisms in ovine immune (IFNγ, IL1B, TNF, and IL4) genes were associated with abortion according to [45]. In Barki sheep, [55] found that differences in the nucleotide sequences of the TLR4 and SOD genes were linked to an increased risk of abortion. In Chinese Holstein cows, [85] reported an association between methylenetetrahydrofolate reductase (MTHFR) gene polymorphism and abortion.

Twenty-five SNPs were found through DNA sequencing of the immune and antioxidant genes; 11 of these SNPs were linked to sheep that were aborted. Six of the SNPs found were synonymous, while five were non-synonymous. The primary source of adaptation and selection is mutation [86]. All of the investigated markers in this case showed exonic region alterations, which resulted in distinct coding DNA sequences in aborted ewes compared to healthy ewes. Non-synonymous mutations modify protein sequences, and animals harboring these mutations are typically targeted by natural selection [86]. Non-synonymous SNP-induced genetic variation modifies the fixed amino acid at the mutant location, which may result in structural and functional abnormalities in the mutated protein [87]. Selection of synonymous mutations was long believed to be either very low or nonexistent [86]. Understanding the physiological differences between healthy and aborted ewes in terms of resistance and vulnerability requires precise molecular characterization of the genes under investigation. Our findings indicate that polymorphisms derived from the ewes’ translated DNA sequence are more valuable than intronic sections.

Substitution mutations such as G214A (D→N) in IL-17, C58T (P→S) and A250G (T→A) in CFH, and C147G (S→R) in SOD3 were predicted to be “probably damaging” because they alter conserved hydrophobic domains that are essential for receptor–ligand binding. They were also predicted to have a moderate effect on protein function. As per the previously mentioned findings. Abortion incidence and proper immunological or antioxidant activity may also be affected by mutations [86,87]. These findings corroborate the links between these SNPs and the likelihood of having an abortion.

It is noteworthy that the expression profile of the investigated markers was elucidated in the course of susceptibility to infectious diseases in livestock. For instance, [55] demonstrated that levels of IL5, IL6, IL1-ß, TNF-α, TLR4, and Tollip were significantly up-regulated in ewes affected with abortion than in resistant ones, while SOD and CAT gene patterns elicited an opposite trend. Compared to resistant kids, kids with diarrhea showed a significant up-regulation of the TMED1, SMURF1, ICAM1, CSF1R, and CFH genes [88]. In addition, [89] cited that mastitic camels were significantly more likely to express the TLR4 and OXSR1 genes. However, the SOD3 and CAT genes elicited a different pattern. In the same vein, [88] mentioned that gene expression levels were considerably higher in endometritis-affected cows than in resistant ones for the TLR4 gene. Furthermore, [90] elaborated that there was considerable up-regulation of the expression level of HMOX1 in endometritis ewes. Moreover, Keap1 and HMOX1 were considerably more highly expressed in diarrheic calves than in resistant ones. The Nrf2 and PRDX2 genes, however, produced a different pattern.

Upon activation, TLR controls the expression of several chemokines and pro-inflammatory cytokines, which aids in further promoting neutrophil recruitment and stimulating both innate and acquired immune responses [91]. These pattern recognition receptor (PRR) SNPs impact an individual’s sensitivity or resistance to mastitis and can cause differences in the host’s response to infections [91]. In inflammatory circumstances, cytokines and NF-kB serve as indirect indicators [92].

The complement system, a part of the body’s immune response that eliminates foreign invaders, triggers an inflammatory response, and removes debris from cells and tissues, is managed by the complement factor H (CFH) genes [93]. Transmembrane P24 trafficking protein 1 (TMED1) is a member of the family of transmembrane proteins containing the emp24 domain, which is involved in the transport of proteins across vesicles [94]. Intercellular adhesion molecule 1 (ICAM-1) or CD54 (Cluster of Differentiation 54) is a protein produced by the ICAM1 gene [95]. ICAM-1 is involved in the T cell-mediated host defense mechanism as well as inflammatory events [95].

SMAD-specific E3 ubiquitin protein ligase 1 (SMURF1) regulates cell motility, polarity, and signaling through the bone morphogenetic signaling system [96]. This gene produces the protein known as colony-stimulating factor 1 receptor (CSF1R), also referred to as CD115 (Cluster of Development 115), and macrophage colony-stimulating factor receptor (M-CSFR), which regulates the development and activity of macrophages [97].

Known as endogenous antioxidant indicators, these activities include the body’s own enzymatic and non-enzymatic antioxidant defenses, such as catalase (CAT) and superoxide dismutase (SOD) [98]. The main inducible defense against oxidative stress is the Keap1-Nrf2 stress response system, which controls the production of cytoprotective genes [99]. Under normal conditions, Keap1 represents a substrate adaptor for cullin-based E3 ubiquitin ligase, which hinders Nrf2 transcriptional action via ubiquitination and proteasomal degradation [100]. This might explain the opposing expression pattern of Keap1 and Nrf2 genes displayed in our investigation.

Hydrogen peroxide (H_2_O_2_) can be catalyzed by the peroxiredoxin (PRDX) family of antioxidant enzyme oxidoreductase proteins thanks to a conserved ionized thiol. By detoxifying peroxides and radicals containing sulphur, thiol-specific peroxidase functions as a sensor for signaling occasions caused by hydrogen peroxide and aids in cell defense against oxidative stress [101]. HMOX1 is also known to be a stress-responsive protein and is thought to have a number of defensive roles in contradiction with different stresses due to its anti-inflammatory, anti-apoptotic, anti-coagulation, anti-proliferative, and vasodilator qualities [102].

While our study demonstrates significant associations between altered expression of immune and antioxidant genes, specific SNPs, and abortion status in Aboudeleik ewes, we recognize that these findings do not establish a direct causal relationship. The significant change in the expression pattern of antioxidant and immune markers in ewes that had abortions could be linked to the overproduction of reactive oxygen species, which damages preimplantation embryos by increasing lipid peroxidation [103] and DNA fragmentation [104], leading often to embryonic death [19,103].

Severe inflammation damages, and the affected tissue, cytotoxic radicals, and pro-inflammatory cytokines are released by the phagocytic cells [105]. Moreover, reactive nitrogen intermediates are important radicals that play a complex role in the inflammatory process [106]. Pregnancy causes marked changes in the regulation of the immune system [45]. Variation in the genes that encode immunity and antioxidant markers may underlie variation in susceptibility to infection by these pathogens, and subsequent abortion, between individuals [45]. Genetic polymorphism could alter the expression of these genes and may mean that these cytokines have altered expression during pregnancy in some individuals, leading to an increased risk of abortion [107].

Several pathogens infect host macrophages, and hence, the immune genes expressed by or acting on macrophages may have some role in abortion related to these infections. It was reported that abortion is associated with infiltrations of CD4+ T cells and NK cells and the subsequent production of IFNG in the placenta. As IFNG enhances NK cell activity and cytotoxic T cell immunity by feedback mechanisms, this can potentially lead to fetal loss or damage [108].

It is possible that a pro-inflammatory response early in gestation protects the dam by eliminating parasites, but leads to destruction of the placental tissues and is thus incompatible with fetal survival [45]. This is supported by findings that pro-inflammatory cytokines are effective at clearing protozoan parasite infections and that their expression increases the likelihood of an unsuccessful pregnancy [109]. The aforementioned reasons could account for the significant amendment in the expression configuration of immune (TLR4, *IL-8*, *IL-17*, *NF-kB*, CFH, TMED1, ICAM1, SMURF1, and CSIFR) and antioxidant (SOD3, CAT, Nrf2, Keap1, PRDX2, and HMOX1) indicators in aborted ewes. Thus, we assume that bacterial etiology is to blame for abortion in the study’s ewes. The abortion group exhibited a substantial inflammatory response, as shown by our real-time PCR data.

### 4.4. Biochemical Profile Changes Linked with Abortion

Studies have varied in the prevalence of different diseases that induce abortion, mostly because of variations in the diagnostic tests used, climate, herd management practices, and sampling techniques [65,110].

The biochemical profiles of the abortion group showed a pronounced inflammatory response, evidenced by elevated serum levels of IL-1α, IL-1β, IL-6, TNF-α, and oxidative markers MDA and NO. Concurrently, a significant decrease in IL-10, antioxidant enzymes (CAT, GSH, GPx), and the pregnancy-related cytokine IFN-τ was observed, suggesting impaired immune regulation and redox homeostasis. The increase in IGFBP1 levels may reflect altered fetal–placental development, consistent with placental dysfunction. Together, these immunological and metabolic disturbances reinforce the hypothesis that both genetic and physiological factors contribute to abortion susceptibility in sheep [23,111].

They become active in order to coordinate the proliferation, migration, and transmission of other immune cells, which work together in order to eliminate invasive germs and restore homeostasis inside the host body. In truth, the attendance of an infective agent and more destructed tissue inside the abortion group generated a more robust immune response and boosted the proinflammatory cytokine reaction in contrast to healthy ewes [112,113]. Additionally, the activation and accumulation of BHBA and NEFA associated with abortion may be caused by negative energy balance and hypoglycemia, which act as an instantaneous stimulus for the release of pro-inflammatory cytokines [114,115]. They seek to rectify this negative energy balance through activation of the adrenal gland and inhibition of pancreatic islets, leading to a pronounced hypercortisolemia and hypoinsulinemia previously documented in analogous circumstances [116]. These results are in agreement with [55,117]. Compared to the control group, the amount of IL-10 in the abortion group was much lower.

The decreased level of IL-10 in the abortion group was in line with earlier research [27,117], which showed that *B. abortus*, a persistent intracellular pathogen, inhibits macrophage immune activation to release IL-10 early during infection. According to in vivo studies [118], mice that lack endogenous IL-10 produce more pro-inflammatory cytokines, which in turn cause the clearance of *B. abortus*.

The current study’s finding that the abortion group had a significantly higher serum IL-6 level suggests the function of these cytokines in the inflammatory response. Given that IL-6 is a primary cause of inflammation [119], the aforementioned findings were in line with prior research conducted by the authors of [55,117,120]. According to recent research, pro-inflammatory cytokines are now more prevalent than anti-inflammatory cytokines in the body’s balance.

The considerable reduction of GPx, GSH, and CAT levels in the abortion group may be ascribed to oxidative stress that occurs during abortion, leading to depletion of the body’s antioxidant resources [121]. According to reports from [117,122,123], the acquired results are consistent with other investigations. Conversely, in the aborted group, the activated pro-inflammatory cytokines increase the liberation of free radicals (MDA and NO). Free radicals are an essential component of the host’s innate defense and cause oxidation of DNA, proteins, lipids, and carbohydrates in microorganisms, which results in damage [124].

According to [125], antioxidants, whether enzymatic or non-enzymatic, neutralize the effects of free radicals and stop them from reacting with the cells in the host body. Unfortunately, in this research, the considerable increase in the serum levels of NO and MDA in the abortion group could be related to the reaction of phagocytic cells to infection [126]. The findings shown in [114,127] were consistent with these findings.

When comparing the blood levels of IFN-τ in the abortion group to the control group, there was a significant decrease. Interferon-tau (IFN-τ) is a new type I interferon that is released by trophoblast cells in ruminants. It binds to type I interferon receptors (IFNARs) on endometrial epithelial cells and promotes signal transduction pathways, helping to initiate pregnancy [128]. Although it is less cytotoxic than other type I interferons, it nevertheless has antiviral and immunomodulatory properties [129]. In diabetic mice that are not fat, interferon-τ can change the immunological profile from a proinflammatory to an anti-inflammatory state, preventing the onset of diabetes [130].

More crucially, IFN-τ released in vivo serves as one of the mediators that provide immunological tolerance to facilitate embryo implantation by inducing an anti-inflammatory response in the bovine uterus [131,132]. Interestingly, in a spontaneous abortion mouse model, the in vivo injection of recombinant IFN-τ can also decrease fetal loss by elevating IL-10 production [133]. According to [134], intramuscular injections of IFN-τ can potentially save embryos that are not developing properly and would otherwise be lost in pregnancy when given to sheep or cows.

Compared to the control ewes, the blood levels of IGFBP1 in the abortion group were significantly (*p* < 0.05) higher. IGFs are endocrine, autocrine, and paracrine stimulators of cell survival, transformation, and mitogenesis. The type 1 IGF receptor (IGF-1R), a tyrosine kinase that mimics the insulin receptor, mediates these effects. Insulin-like growth factor-binding proteins (IGFBPs) regulate the availability of free IGF for interaction with IGF-1R. IGFBP proteases, which alter the relative affinities of IGFBPs, IGFs, and IGF-1R, also control IGFBP activity [135]. IGFBP-1 is only present in the luminal epithelium of the uterus and is thought to be crucial in controlling the movement of IGFs from the endometrium to the uterine lumen [136]. Furthermore, IGFBP-1 is a key carrier protein in fetal serum and the predominant binding protein in amniotic fluid. During pregnancy, its concentrations in the mother’s circulation increase [137]. IGFBP-1 has been linked to the anomalies in placental development that are seen in preeclampsia in humans [138].

### 4.5. Relevance of Findings to Vaccination Strategies

The present findings offer valuable insights that may inform the optimization of vaccination strategies against abortion-causing bacterial pathogens in sheep. The identification of *Brucella melitensis*, *Salmonella* spp., and *Campylobacter* spp. as major causative agents reinforces the necessity of targeted vaccination programs tailored to regional pathogen prevalence. These bacteria are known to evade host immunity, and the current study shows that they elicit strong inflammatory and oxidative responses, as evidenced by elevated pro-inflammatory cytokines, immune gene expression, and oxidative stress markers.

Importantly, the observed genetic polymorphisms in immune and antioxidant-related genes suggest that some animals may have inherently weaker immune responses to infection, potentially influencing vaccine responsiveness. This highlights the need for integrated control programs that consider both genetic background and vaccine efficacy. In populations with high abortion rates, identifying genetically susceptible individuals could help enhance vaccine coverage effectiveness by prioritizing booster schedules or multivalent vaccines that provide broader protection.

Furthermore, since excessive inflammation and oxidative stress were hallmarks of the aborted cases, adjuvants or formulations that promote a balanced Th1/Th2 immune response without excessive immunopathology may be more beneficial. Monitoring post-vaccination immune markers (e.g., IL-6, TNF-α, and IFN-τ) could also serve as indicators of successful immunization and fetal protection.

### 4.6. Potential for Diagnostic Kit Development

Although this study identified several promising genetic and biochemical markers associated with abortion in ewes, a commercially available diagnostic kit based on these findings is not yet available. Nevertheless, the molecular targets and serum markers identified herein, such as non-synonymous SNPs in immune and antioxidant genes, altered expression levels of IL-17, Nrf2, SOD3, and elevated levels of IL-1β, MDA, and IFN-τ represent strong candidates for diagnostic assay development.

The future goal is to translate these findings into a field-deployable, rapid, and cost-effective molecular diagnostic kit capable of identifying genetically susceptible animals or monitoring abortion risk in breeding flocks. Such a tool could also aid in evaluating vaccine responsiveness or early detection of subclinical infections. To achieve this, further validation across broader populations and environmental settings is required. Our team is actively seeking collaboration with biotech partners and diagnostic companies to support kit prototyping and potential commercialization. By bridging the gap between molecular research and farm applications, this approach could significantly improve reproductive management and disease control in sheep.

The present study contributes novel insights to the international veterinary literature through a multifaceted investigation of bacterial abortion in Aboudeleik sheep. This is the first study to integrate pathogen identification, serum cytokine and oxidative marker profiling, transcript-level gene expression, and SNP analysis in this breed. By identifying specific genetic polymorphisms associated with abortion susceptibility, this research provides a foundation for future selective breeding programs aimed at improving reproductive resilience. These findings are particularly valuable for small ruminant production systems in arid and resource-limited settings and contribute to broader One Health goals, given the zoonotic nature of the identified pathogens.

The findings of this study have practical significance for improving reproductive health and productivity in local Aboudeleik sheep populations. By identifying specific immune and antioxidant gene polymorphisms associated with abortion susceptibility, this research provides a scientific foundation for the development of marker-assisted selection (MAS) strategies. Such tools can enable early identification and culling or selective breeding of animals genetically predisposed to miscarriage, thereby improving lambing rates and reducing economic losses for smallholder farmers. This is particularly relevant for communities in arid and resource-limited regions where veterinary infrastructure is limited and abortion-related losses can significantly affect household income. The integration of SNP screening into national or regional breeding programs, alongside improved biosecurity and vaccination strategies, could substantially enhance flock resilience and support sustainable livestock production in rural areas.

The practical application of SNP-based selection in the Aboudeleik sheep population, while promising, is currently constrained by factors such as limited genotyping infrastructure, absence of centralized breeding records, and small flock sizes in rural settings. However, a multi-phase implementation strategy can address these challenges. First, the SNP markers identified in this study, particularly those with significant allele frequency differences and functional implications (e.g., IL-17 G214A, CFH A250G, SOD3 C147G), should be validated in larger, geographically diverse cohorts of Aboudeleik ewes to confirm their association with abortion susceptibility. Second, functional studies can be conducted to assess the biological relevance of these SNPs. Third, cost-effective genotyping platforms, such as TaqMan or KASP assays, could be developed for use in marker-assisted selection (MAS) within nucleus flocks. Finally, integrating these markers into existing breeding schemes can enhance the genetic resilience of the breed against reproductive losses. While implementation will require capacity building and institutional support, our findings provide a foundation for precision breeding strategies in this economically important local breed.

Although the current study provides valuable insights into the immunological and antioxidant changes associated with abortion in sheep, we acknowledge certain limitations. The case control design does not allow for the determination of causality, and the sample size, while appropriate for an exploratory study, may not capture all biological variability present in larger flocks. Furthermore, the use of naturally occurring abortion cases may introduce unrecognized confounders, despite efforts to control environmental and management factors. Future studies incorporating controlled experimental infections, longitudinal monitoring, and multi-breed comparisons will be essential to validate and expand upon these findings. Nevertheless, the integration of bacteriological, molecular, and biochemical analyses in this study offers a comprehensive approach to understanding the multifactorial etiology of abortion in sheep. We acknowledge that the number of aborted cases in this study (n = 37) may limit the generalizability of the findings. However, the consistency of the molecular and biochemical trends observed, supported by statistical analysis, provides a valid basis for preliminary conclusions. Future studies with larger sample sizes and multi-farm representation are planned to validate and expand upon these results.

## 5. Conclusions

This study provides significant added value to the international veterinary field by linking bacterial pathogens, host genetic variation, and biochemical markers to abortion in sheep. The high prevalence of *Brucella melitensis*, *Salmonella* spp., and *Campylobacter* spp. in aborted cases underscores the need for targeted biosecurity and immunization measures in sheep flocks. The identified SNPs and altered expression patterns in immune and antioxidant genes represent promising biomarkers for diagnostic and breeding strategies. These alterations were accompanied by elevated levels of serum cytokines and oxidative stress markers, indicating a complex interplay between genetic predisposition, immune activation, and oxidative damage in the development of abortion. Furthermore, the integrated approach of correlating field-isolated zoonotic pathogens with host susceptibility aligns with global efforts to improve livestock productivity and public health under the One Health framework. Importantly, these insights carry significant implications for the design and timing of vaccination programs.

## Figures and Tables

**Table 1:** Primer sequences, target genes, amplicon sizes, and cycling conditions.

Target Gene	Primers Sequences	Amplified Segment (bp)	Primary Denaturation	Amplification (35 Cycles)			Final Extension	Reference
				Secondary Denaturation	Annealing	Extension		
*Salmonella invA*	GTGAAATTATCGCCACGTTCGGGCAA	284	94 °C 5 min	94 °C 30s	55 °C 30 s	72 °C 30 s	72 °C 7 min.	[38]
	TCATCGCACCGTCAAAGGAACC							
*L. monocytogenes 16S rRNA*	ggA CCg ggg CTA ATA CCg AAT gAT AA	1200	94 °C 5 min	94 °C 30 s	60 °C 40 s	72 °C 1 min.	72 °C 10 min.	[39]
	TTC ATg TAg gCg AgT TgC AgC CTA							
*Campylobacter * *23S rRNA*	TATACCGGTAAGGAGTGCTGGAG	650	94 °C 5 min	94 °C 30 s	55 °C 40 s	72 °C 45 s	72 °C 10 min.	[40]
	ATCAATTAACCTTCGAGCACCG							
*Leptospira secY*	GCGATTCAGTTTAATCCTGC	202	95 °C 5 min	94 °C 30 s	54 °C 30 s	72 °C 30 s	72 °C 7 min.	[41]
	GAGTTAGAGCTCAAATCTA							
*Brucella IS711*	GGC-GTG-TCT-GCA-TTC-AAC-G	839	95°C 5 min	94 °C 30 s	55 °C 40 s	72 °C 50 s	72 °C 10 min.	[42]
	GGC-TTG-TCT-GCA-TTC-AAG-G							
*Chlamydia psittaci pmp*	ATGAAACATCCAGTCTACTGG	300	94 °C 5 min	94 °C 30 s	50 °C 30 s	72 °C 30 s	72 °C 7 min.	[43]
	TTGTGTAGTAATATTATCAAA							
*Coxiella burneti ISIIII*	TAT GTA TCC ACC GTA GCC AGT C	687	94 °C 5 min	Five cycles at 94 °C for 30 s, 66 to 61 °C (the temperature was decreased by 1 °C between consecutive steps) for 1 min, and 72 °C for 1 min. These cycles were followed by 35 cycles consisting of 94 °C for 30 s, 61 °C for 30 s, and 72 °C for 1 min.			72 °C 7 min	[44]
	CCC AAC AAC ACC TCC TTA TTC							

**Table 2:** Real-time PCR primers made of oligonucleotides that are forward and reverse for immune and antioxidant genes under study.

Investigated Marker	Primer	Product Size (bp)	Annealing Temperature (°C)	GenBank Isolate
*TLR4*	F5′-ATGATGGCGCGTGCCCGCCG-3 R5′-GTCTCCACGGCCACCAGCTTC-3′	407	58	NM_001135930.1
*IL-8*	F5′-CGAGAAGTCCTCTGGGACAGC-3 R5′-CATGGATCTTGCTTCTCAGCTC-3′	389	60	X78306.1
*IL-17*	F5′-ATCTACAGTGAACTGGAAGGAG-3′ R5′-CGAAGGACCAGGATCTCTTGCT-3′	389	60	XM_004018887.6
*NF-kB*	F5′-ATCCACCTGCACGCACACAGC-3′ R5′-GCTGTCATAGATGGCGTCCGAC-3′	411	60	XM_060416845.1
*CFH*	F5′-GGGCCTCCTCCACCAATAGAC-3′ R5′-CTTTCCTTCCCGACAAGTTGT-3′	339	58	EU888587.1
*TMED1*	F5′-AGCACTGGCTGGCTTGCAGGT-3′ R5′-GTGACTGTTCTGGCAAGAACAC-3′	408	58	XM_060415483.1
*ICAM*	F5′-TGAGAGTGAACTGCAGTATC-3′ R5′-CTCGGAGCAGCACCATGGAGA-3′	324	58	AF110984.1
*SMURF1*	F5′-AAGATCCGTCTGACAGTATTA-3′ R5′-CCTGCAGTCCACCACAGAGCCG-3′	420	60	XM_027961801.3
*CSFIR*	F5′-GTGTCTGAGAATCCGGCTCTCT-3′ R5′-TCTCCAGGCTCAGTGCAGCGGT-3′	335	58	XM_027970397.2
*SOD3*	F5′-ATCCGCGACATGCACGCCAAG-3′ R5′-CCAGACCTGGCCATCTCGCAC-3′	381	60	XM_027970902.2
*CAT*	F5′-CTGATGTCCTGACCACTGGCGC-3′ R5′-CATGTCCGGATCCTTCAGGTG-3′	473	58	XM_060400055.1
*Nrf2*	F5′-CCGCTGCTCCTCTGCTCAAGA-3′ R5′-CAGCTCATGCTCCTTCTGTCGT-3′	416	58	OR900054.1
*Keap1*	F5′-GCTCGGTGCGCGGGTGGCAC-3′ R5′-TGGCTGAGCCGCAGTTCATTCA-3′	360	60	XM_027969637.3
*PRDX2*	F5′-ATGGCCTGCGGCAAGGCGCAC-3′ R5′-TCATCTTCCTTCAGCACGCCAT-3′	365	58	NM_001166200.1
*HMOX1*	F5′-CAGAGGAGCTGCACCGCCGGG-3′ R5′-ACAGCTGGATGTTGAGCAGGA-3′	402	60	OR900057.1
*ß. actin*	F5′-AATTCCATCATGAAGTGTGAC-3′ R5′-GATCTTGATCTTCATCGTGCT-3′	150	58	KU365062.1

**Table 3:** Prevalence of bacteria isolated from aborted fetuses, vaginal swabs, and placentas of aborted ewes.

Microorganisms	Sites of Isolation						Total Isolates (n = 111)	
	Aborted Fetuses (n = 37)		Vaginal Swab (n = 37)		Placenta Swab (n = 37)			
*Bacterial isolates*	No.	%	No	%	No	%	No	%
*Brucella melitensis*	5	13.5	3	8.1	4	10.8	12	10.8
*Salmonella*	4	10.8	1	2.7	3	8.1	8	7.2
*Campylobacter* sp.	3	8.1	1	2.7	2	5.4	6	5.4
*Listeria monocytogens*	0	0	0	0	0	0	0	0
*Coxiella burnetii*	0	0	0	0	0	0	0	0
*Chlamydia psittaci*	0	0	0	0	0	0	0	0
*Total bacterial isolates*	12	32.4	5	13.5	9	24.3	26	23.4

**Table 4:** Bacteria isolated from aborted sheep fetuses, categorized by their sites of isolation from the internal organs.

Microorganisms	Total Isolates		Sites of Isolation							
			Stomach Content		Liver		Spleen		Lungs	
*Bacterial isolates*	No	%	No	%	No	%	No	%	No	%
*Brucella melitensis*	5	13.5	5	100	4	80	4	80	3	60
*Salmonella*	4	10.8	4	100	3	75	3	75	2	50
*Campylobacter* sp.	3	8	3	100	2	66.6	2	66.6	1	33.3
*Listeria monocytogens*	0	0	0	0	0	0	0	0	0	0
*Coxiella burnetii*	0	0	0	0	0	0	0	0	0	0
*Chlamydia psittaci*	0	0	0	0	0	0	0	0	0	0
*Total bacterial isolates*	12	32.4	12	100	9	75	9	75	6	50

**Table 5:** Dispersion of immune markers in aborted and control groups with a single base differential and a potential genetic alteration.

Gene	SNP	Healthy n = 43	Aborted n = 37	Total n = 80	Chi-Square Value X^2^	*p*-Value	Kind of Inherited Change	Amino Acid Order and Sort
TLR4	A90C	25/43	-/37	25/80	31.2	0.001	Synonymous	30 V
	G297A	31/43	-/37	31/80	43.5	0.001	Synonymous	99 Q
IL-8	C129T	18/43	-/37	18/80	19.9	0.001	Synonymous	43 L
	C238T	-/43	21/37	21/80	33	0.001	Synonymous	80 L
IL-17	G90A	26/43	-/37	26/80	33.1	0.001	Synonymous	30 V
	G156A	-/43	28/37	28/80	50	0.001	Synonymous	52 Q
	G214A	-/43	22/37	22/80	35.2	0.001	Non-synonymous	72 D to N
NF-kB	A264G	23/43	-/37	23/80	27.7	0.001	Synonymous	88 K
CFH	C58T	36/43	-/37	36/80	56.3	0.001	Non-synonymous	20 P to S
	A250G	-/43	22/37	22/80	35.2	0.001	Non-synonymous	84 T to A
TMED1	T337C	26/43	-/37	26/80	33.1	0.001	Non-synonymous	113 W to L
ICAM	C88T	19/43	-/37	19/80	21.4	0.001	Synonymous	30 P to S
SMURF1	T120G	31/43	-/37	31/80	43.5	0.001	Synonymous	40 T
	T198C	-/43	28/37	28/80	50	0.001	Synonymous	66 S
	C369T	-/43	22/37	22/80	35.2	0.001	Synonymous	123 S
CSFIR	T52C	-/43	16/37	16/80	23.2	0.001	Non-synonymous	18 Y to H
	A132G	32/43	-/37	32/80	45.8	0.001	Synonymous	44 P

**Table 6:** Dispersion of antioxidant markers in aborted and control groups with a single base differential and a potential genetic alteration.

Gene	SNP	Healthy n = 43	Aborted n = 37	Total n = 80	Chi-Square Value X^2^	*p*-Value	Kind of Inherited Change	Amino Acid Order and Sort
SOD3	C147G	-/43	22/37	22/80	35.2	0.001	Non-synonymous	49 S to R
CAT	C155T	19/43	-/37	19/80	21.4	0.001	Non-synonymous	52 T to M
Nrf2	C179T	-/43	24/37	24/80	39.8	0.001	Non-synonymous	60 T to I
Keap1	C114T	-/43	15/37	15/80	21.4	0.001	Synonymous	38 G
	G268A	29/43	-/37	29/80	39.1	0.001	Non-synonymous	90 A to T
PRDX2	T237G	-/43	22/37	22/80	33	0.001	Synonymous	79 S
HMOX1	T99C	31/43	-/37	31/80	43.5	0.001	Synonymous	33 S
	C284T	19/43	-/37	19/80	21.4	0.001	Non-synonymous	95 A to V

**Table 7:** Dispersion of immune and antioxidant markers in aborted and healthy ewes with a single base differential and a potential genetic alteration.

		Predicted Group Membership		Total
		Healthy	Abortion	
Count	Healthy	37	0	100
	Diseased	0	43	100
%	Healthy	37	0.0	100.0
	Diseased	0.0	43	100.0

**Table 8:** Comparison between the immunological marker concentrations in the control and aborted groups. Values are mean ± SD.

Parameters	Control Group	Abortion Group	*p*-Value
IL-1α (Pg/mL)	7.1 ± 0.8	34.9 ± 4.2 *	0.003
IL-1β (Pg/mL)	9.3 ± 1.8	68.1 ± 6 *	0.001
IL-6 (Pg/mL)	2.9 ± 0.4	21.2 ± 2 *	0.001
TNF-α (Pg/mL)	5 ± 0.4	8.1 ± 0.6 *	0.02
IL-10 (Pg/mL)	29.8 ± 3.1	9.8 ± 1.1 *	0.004
(IFN-τ) (ng/mL)	4.7 ± 1.3	0.7 ± 0.1 *	0.04

MDA: malondialdhyde; NO: nitric oxide; CAT: catalase; GPx: glutathione peroxidase; GSH: glutathione reductase; IGFBP1: insulin-like growth factor binding proteins1; (IFN-τ): interferon tau. * *p* < 0.05.

**Table 9:** Comparison between the oxidative stress and hormonal marker concentrations in the control and aborted groups. Values are mean ± SD.

Parameters	Control Group	Abortion Group	*p*-Value
MDA (nmol/mL)	6.8 ± 0.8	17.3 ± 1.5 *	0.004
NO (μmol/L)	7.4 ± 0.7	17.1 ± 1.4 *	0.004
CAT (U/L)	37.8 ± 2.6	18.8 ± 1.3 *	0.003
GPx (U/mL)	60.5 ± 3.5	40 ± 0.5 *	0.004
GSH (mg/dL)	42.6 ± 2.6	26 ± 0.5 *	0.003
Progesterone (ng/mL)	1.53 ± 0.2	0.33 ± 0.1 *	0.007
IGFBP1 (ng/mL)	50 ± 2.8	63.3 ± 2 *	0.01

Asterisks (*) indicate statistically significant differences (* *p* < 0.05).

## Data Availability

The data are contained within this paper and Supplementary Materials.

## Supplementary Materials

The following supporting information can be downloaded at: https://www.mdpi.com/article/10.3390/vetsci12080719/s1, Figure S1. (A, B, C): Electrophoresis analysis of PCR product of amplified (A) *Brucella melitensis*, (B) *Salmonella* spp. (C) *Campylobacter* spp. (A) Lane1,2,3 and 5 indicate a positive amplification of *Brucella melitensis* at the 839, (B) Lanes 1,2 and 4 indicate positive amplification of *Salmonella* at the 284 bp and (C ) All samples indicate positive amplification at 650bp for *Campylobacter*. Figure S2. (A, B, C, D) Electrophoresis analysis of PCR product of amplified (A) *Coxiella burnetii*, (B) *Chlamydia psittaci*, (C) *Listeria monocytogens* and (D) *Leptospira*. All sample negative for all bacteria at 687, 300, 1200 and 202 bp, respectively.
