# Supplementary material for: Individual Genomic Loci, Transcript Levels, and Serum Profiles of Immune and Antioxidant Markers Associated with Bacteria-Induced Abortion in Sheep (Ovis aries)
Source: Vet Sci. 2025 Jul 31;12(8):719. doi: 10.3390/vetsci12080719 (PMC12390017; doi:10.3390/vetsci12080719)
Supplement: Supplementary file 1 [file vetsci-12-00719-s001.zip › vetsci-3760410-supplementary.pdf]

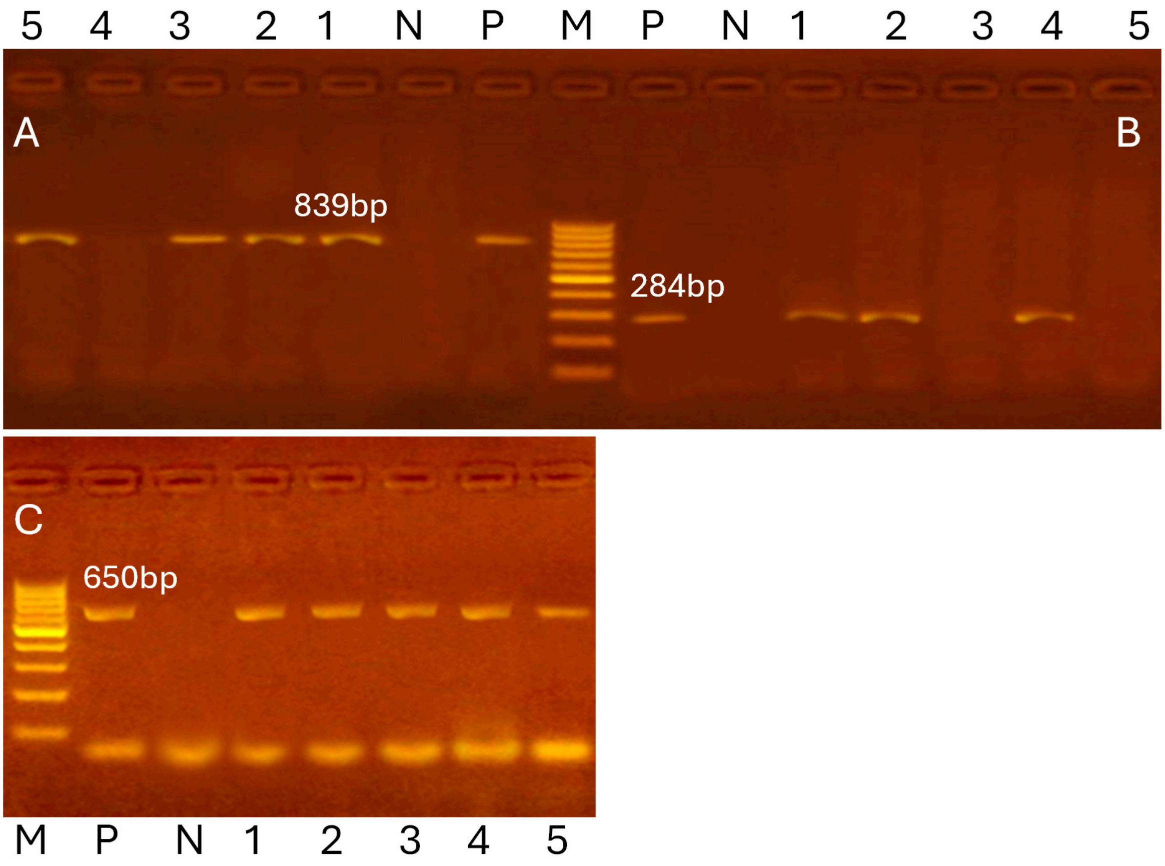

Figure S1. (A, B,C): Electrophoresis analysis of PCR product of amplified (A) *Brucella melitensis*, (B) *Salmonella* spp. (C) *Campylobacter* spp. (A) Lane1,2,3 and 5 indicate a positive amplification of *Brucella melitensis* at the 839, (B) Lanes 1,2 and 4 indicate positive amplification of *Salmonella* at the 284 bp and (C) All samples indicate positive amplification at 650bp for *Campylobacter*.

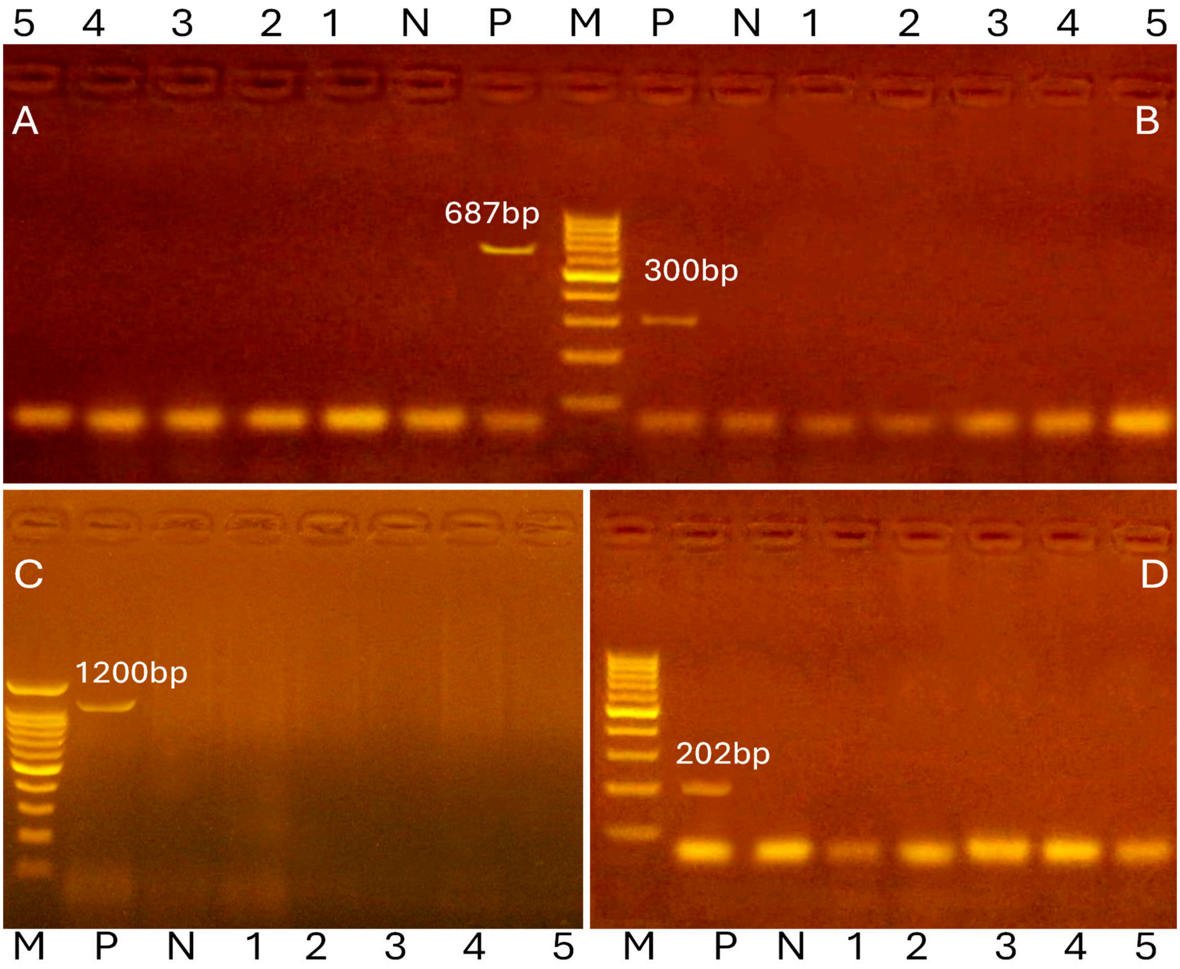

Figure S2. (A, B, C, D) Electrophoresis analysis of PCR product of amplified (A) *Coxiella burnetii*, (B) *Chlamydia psittaci*, (C) *Listeria monocytogens* and (D) *Leptospira*. All sample negative for all bacteria at 687, 300, 1200 and 202 bp, respectively.
